# Introduction of Sustained Release Opipramol Dihydrochloride Matrix Tablets as a New Approach in the Treatment of Depressive Disorders

**Published:** 2006-12

**Authors:** Ümit Gönüllü, Melike Üner, Gülgün Yener, Turan Altınkurt

**Affiliations:** *Istanbul University, Faculty of Pharmacy, Department of Pharmaceutical Technology, Beyazıt 34119 Istanbul, Turkey*

**Keywords:** compression parameters, controlled drug delivery, direct compression, matrix tablets, opipramol 2-HCl, release kinetics, sustained drug release

## Abstract

Opipramol 2-HCl (OP) is used for therapy of general somatoform and anxiety disorders. Conventional tablets in the market contain 50 mg OP to be used once or up to three times a day in effective treatment of depression in mild. In case of serious depressive disorders, OP may be administired up to 300 mg a day. Decrease in frequency of high dose administration via sustained drug release would reduce incidence of symptoms of intoxication in long-term use of OP. With this aim, OP matrix tablets containing 100 mg were prepared by direct compression method to be used once a day to provide patient compliance and constant blood level, consequently to decrease side effects. Two concentrations of polymers (10% and 20%): hydroxypropylcellulose (HPC) and hydroxypropyl methylcellulose (HPMC), sodium alginate (NaAlg), xanthan gum (XG) and Carbopol^®^941 (C941) were used in preparation of matrix tablets. Drug release study were performed in distilled water, pH1.2 HCl buffer and pH7.4 phosphate buffer solutions according to the Method II in USP 29. Two commercial tablets containing 50 mg OP available in Turkish market were used for comparison. Kinetic models of release patterns from tablets were evaluated. Drug release was displayed slower to faster pattern in order of formulations containing C941, HPMC and HPC. Drug release was significantly faster in tablets of 10% polymers than those of 20%. NaAlg and XG were insufficient to sustain drug release. The most sustaining drug release effect at the lowest polymer concentration was obtained with C941. Drug release from matrix tablets containing 10% C941 was determined as 58.2%, 52.4 and 57.0% in related dissolution mediums above after 8 hours, respectively. However, HPMC and HPC sustained drug release at 20% concentration. As a result, Carbopol® 941, HPMC and HPC can be suggested as suitable to prepare matrix tablets of OP.

## INTRODUCTION

Antidepressant therapy has been paid great attention especially in the last decade. Opipramol 2-HCl (4-[3-(5H-dibenz[b,f]-azepine-5-yl)-propyl]-1-piperazine-ethanol dihydrochloride, CAS 315-72-0) is an atypical anxiolytic and antidepressive drug. It is a psychotropic drug commonly used for therapy of anxious-depressive states, somatoform disorders and general anxiety disorders (1-4). Although it is structurally very similar to imipramine, it does not represent a tricyclic antidepressant drug as it does not inhibit the neuronal uptake of norepinephrine and/or serotonin. It was reported to be a rather potent sigma ligand with high affinity to sigma1 and lower affinity to sigma2 sites. Sigma2 sites define the anxiolytic properties of OP (5, 6). INSIDON^®^ is the most well-known and commonly used commercial preparation of OP. Sugar-coated and film-coated tablets of OP are available in the market of Europe and Türkiye (7-9).

OP is given orally in doses of 50 mg to 300 mg divided daily. It is completely absorbed from the gastro-intestinal tract. Its terminal plasma half-life is 6-11 hours (3, 4, 9, 10). In spite of widespread use of antidepressants, an increased incidence of acute overdoses have been reported (11, 12). OP may develop the symptoms of intoxication in regular long-term use. The symptoms of intoxication are usually non-specific, with excitation or unconsciousness, convulsions, blood pressure changes, and cardiac effects such as taschycardia, atrial fibrillation, conduction disturbances and arrhythmias. It was reported that a fatal outcome appeared to be related to decreased cardiac output and hypotension, culminating in death from cardiac collapse (12, 13).

In recent years, increasing attention is being given for administering drugs in a controlled manner for better therapeutic end point. Various controlled release dosage forms have been developed or are still under development with this aim. Their advantages over other conventional dosage forms have been reported to maintain relatively constant therapeutic blood levels of the drug for a desired period, for years (14-17). Matrix tablets are one of oral controlled release drug delivery systems (18-20).

Conventional tablets in the market contain 50 mg OP to be used once or up to three times a day in effective treatment of depression in mild. A sustained drug delivery system of OP is not available in the market all around the world. Various patented inventions on controlled drug delivery for oral administration indicate that OP also can be formulated for sustained drug release (21-23). Therefore, in this study, we decided 100 mg dose for matrix OP tablets to be used once a day to provide constant blood level and decrease in side effects. Tablets were prepared using cellulose derivatives (HPC and HPMC), sodium alginate (NaAlg), xanthan gum (XG) and Carbopol^®^941 (high molecular weight homo- and copolymers of acrylic acid crosslinked with a polyalkenyl polyether) (C941) at 10% and 20% concentrations by direct compression method. Compression parameters including weight variation, diameter and height, hardness and friability (%) of tablets were tested in order to guarantee physical qualification of tablets. Then, *in vitro* dissolution study was performed in three dissolution mediums (i.e. distilled water, pH1.2 HCl and pH7.4 phosphate buffer solutions). Kinetics of drug release from the tablets were determined. Additionally, same studies were repeated on a conventional tablet (A) and a light protective film-coated tablet (B) of 50 mg OP which are available in the Turkish market for comparison.

## MATERIALS AND METHODS

### Chemicals

Opipramol 2-HCl was obtained from Deva Holding (Türkiye). HPC (Nisso HPC-H) and HPMC (Methocel K15 Premium EP) were kindly provided from Nippon (Japan) and Colorcon (England), respectively. Sodium alginate, xanthan gum and Carbopol^®^ 941 were purchased from Sigma (Germany), Selectchemie AG (Switzerland) and BF Goodrich (USA), respectively. Lactose anhydrous (DMV-The Netherlands) and magnesium stearate (Prever, Italy) were obtained from Liba Laboratuarları A.Ş. All other chemicals were of analytical grade.

### Preparation of opipramol matrix tablets

All matrix tablets were prepared by direct compression method. Constituents of tablet formulations are seen in Table 1. Powder mixtures were mixed in a planetary mixer and were then compressed by using a hydrolic press (Yeniyurt, Türkiye) equipped with 12.5 mm diameter flat punch under 5000-5500 psi pressure.

**Table 1:** Composition of the matrix tablets

Constituents (%)	Formulations									
	1	2	3	4	5	6	7	8	9	10

OP	20	20	20	20	20	20	20	20	20	20
Lactose anhydrous	70	70	70	70	70	60	60	60	60	60
HPC	10	-	-	-	-	20	-	-	-	-
HPMC	-	10	-	-	-	-	20	-	-	-
NaAlg	-	-	10	-	-	-	-	20	-	-
XG	-	-	-	10	-	-	-	-	20	-
C941	-	-	-	-	10	-	-	-	-	20
Magnesium stearate	0.5	0.5	0.5	0.5	0.5	0.5	0.5	0.5	0.5	0.5

### Investigation of compression parameters

Weight variation (tablet uniformity), diameter and thickness, hardness and friability of tablets were investigated with 20 tablets of each formulation except hardness test. 10 tablets were used for hardness test. Weight variation was determined according to USP 29. Diameter and thickness of tablets were measured by using a micrometer (Mitutoyo, Japan). Hardness test was determined with a Monsanto tablet hardness tester (Italy). In friability test, tablets were weighed (W_1_) and rotated at 25 rpm for 4 min in an Aymes friabilitor (Türkiye). Tablets were reweighed (W_2_) and friability % (F%) was calculated with the equation below:

(a) $$F\%=\left[\left(W_{1}-W_{2}\right)/W_{1}\right]\cdot 100$$

### *In vitro* dissolution studies

Drug release from matrix tablets were examined by using a dissolution apparatus (Aymes, Türkiye) in 900 ml of various mediums (distilled water, pH1.2 HCl and pH7.4 phosphate buffer solutions) at 37 ± 0.5°C according to the Method II (Paddle) in USP 29. Rotation speed of paddle was 50 rpm. 1 ml samples were taken at determined time intervals, diluted to 25 ml and filtered through S&S^5893^ type blue ribbon filter paper. Opipramol concentrations in filtered samples were determined with a Shimadzu UV-1601 Spectrophotometer (Japan) at 254 nm against corresponding aqueous medium. Amounts of drug present in the samples were calculated with the help of appropriate calibration curves constructed from reference standarts in the range of 2-12 mcg/ml. Drug dissolved at specified time periods was plotted as percent release versus time (hours) curve. Data were statistically evaluated using GraphPad Prism-[one-way ANOVA] statistical programme and release kinetics of drug from matrix tablets were determined.

## RESULTS AND DISCUSSIONS

### Compression parameters

Data obtained from physical controls of matrix tablets met the compendial requirements. Weight of tablets was in a range of 491.3 mg and 509.5 mg indicating homogenous drug content in tablets. Diameter of all tablets was 1.280 cm, but their heights were in a range of 0.295 cm and 0.305 cm. Hardness values were in the range of 9.89 kp and 12.01 kp. All tablets displayed friability lower than 0.5%.

### Dissolution studies and mechanism of drug release

10% polymer concentration generally gave faster drug release compared to 20% for all the tablet formulations in water, pH1.2 HCl buffer and pH7.4 phosphate buffer solutions as expected.

**Figure 1:**
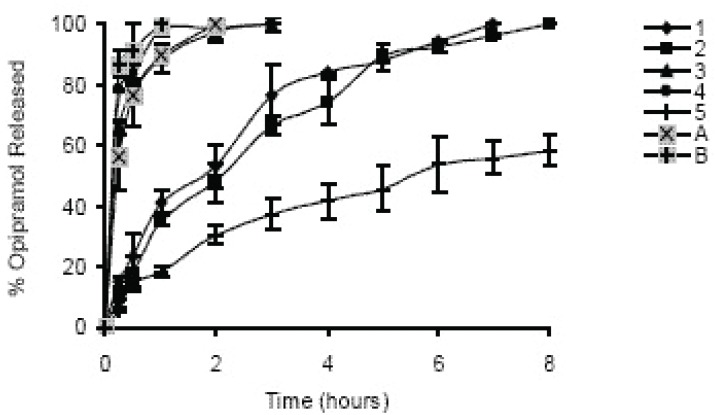
Release profiles of drug from the matrix tablets containing 10% HPC, HPMC, NaAlg, XG and C941 (Formulations 1, 2, 3, 4 and 5, respectively) with commercial tablets A and B in distilled water (n=6).

**Figure 2:**
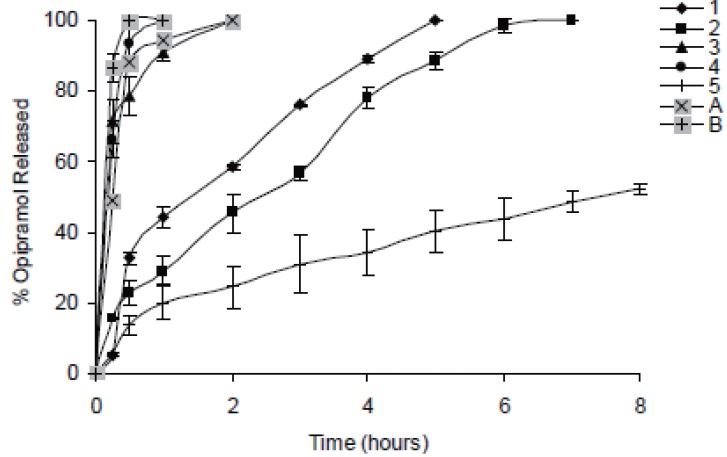
Release profiles of drug from the matrix tablets containing 10% HPC, HPMC, NaAlg, XG and C941 (Formulations 1, 2, 3, 4 and 5, respectively) with commercial tablets A and B in pH1.2 HCl buffer solution (n=6).

**Figure 3:**
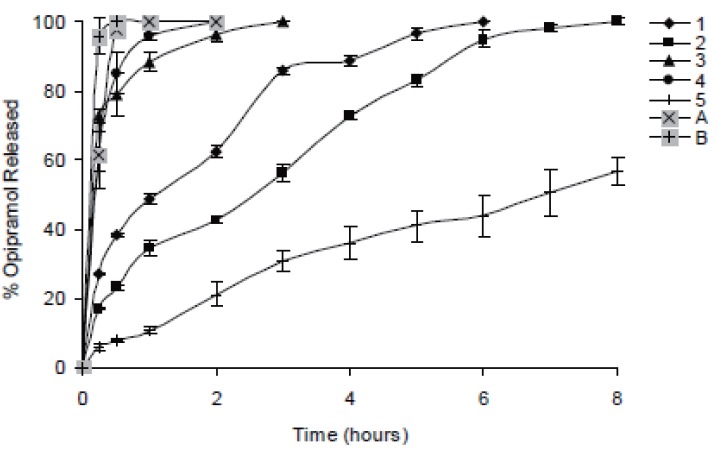
Release profiles of drug from the matrix tablets containing 10% HPC, HPMC, NaAlg, XG and C941 (Formulations 1, 2, 3, 4 and 5, respectively) with commercial tablets A and B in pH 7.4 phosphate buffer solution (n=6).

**Figure 4:**
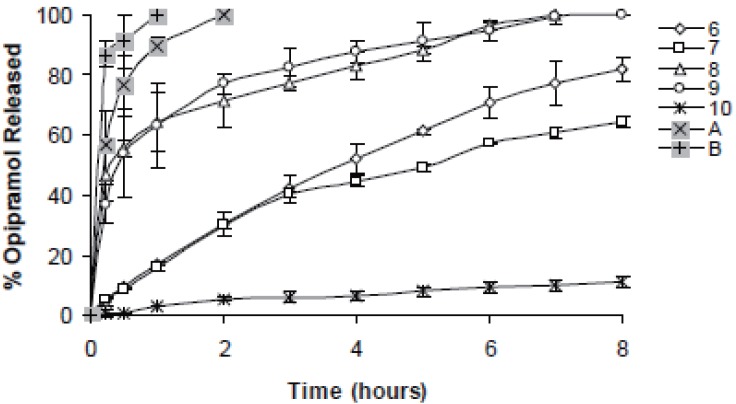
Release profiles of drug from the matrix tablets containing 20% HPC, HPMC, NaAlg, XG and C941 (Formulations 6, 7, 8, 9 and 10, respectively) with commercial tablets A and B in distilled water (n=6).

**Figure 5:**
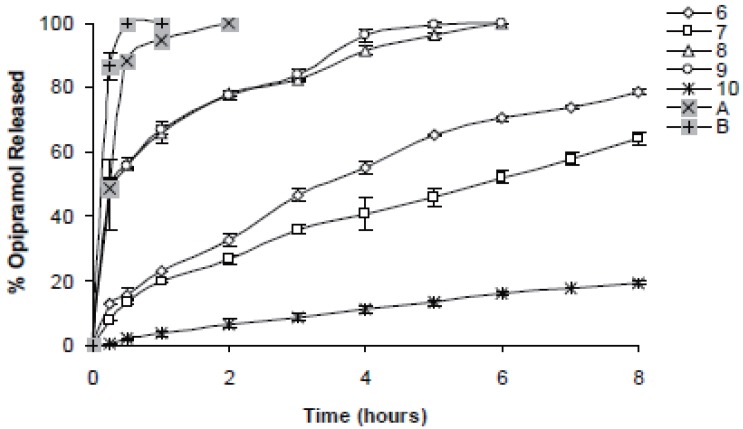
Release profiles of drug from the matrix tablets containing 20% HPC, HPMC, NaAlg, XG and C941 (Formulations 6, 7, 8, 9 and 10, respectively) with commercial tablets A and B in pH1.2 HCl buffer solution (n=6).

**Figure 6:**
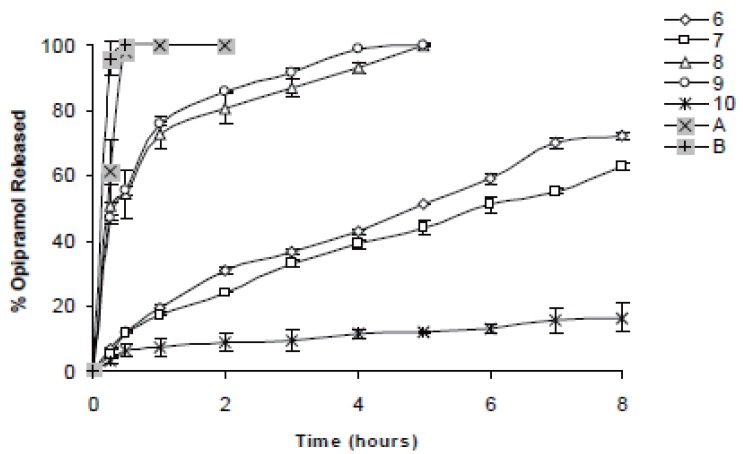
Release profiles of drug from the matrix tablets containing 20% HPC, HPMC, NaAlg, XG and C941 (Formulations 6, 7, 8, 9 and 10, respectively) with commercial tablets A and B in pH 7.4 phosphate buffer solution (n=6).

In case of 10%, slowest drug release was observed with C941 (Formulation 5) followed by HPMC, HPC, Na Alg and XG, respectively (Formulations 2, 1, 3 and 4) (Figures 1-3). In case of 20%, drug release followed the same formulation order (Formulations 10, 7, 6, 8 and 9) (Figures 4-6). NaAlg and XG were not sufficient to sustain drug release compared to the other polymers and difference in their drug release patterns were statistically non-significant as can be seen in Table 2. Significant differences were found in the release profiles of all the other formulations in three mediums (Table 2). Matrix tablets of HPMC and HPC (Formulations 2 and 1) at 10% concentration released 100 % of drug before the 8^th^ hour. In case of 20% concentration, 64.5%, 64.2% and 62.7% of drug was released with HPMC (Formulation 7) while 81.8%, 78.8% and 77.4% was released with HPC (Formulation 6) in dissolution mediums, respectively. 10% C941 (Formulation 5) released 58.2%, 52.4% and 57.0% of drug after 8 hours as 20% C941 (Formulation 10) released 11.2%, 19.1% and 16.5%, respectively. Commercial tablets A and B released 100% drug content within 2 and 1 hours, respectively.

**Table 2:** Statistical evaluations of drug release from the matrix tablets in various dissolution mediums

Formulations	Dissolution Mediums			Formulations	Dissolution Mediums		
	Distilled water	pH1.2 HCl buffer	pH7.4 phosphate buffer		Distilled water	pH1.2 HCl buffer	pH7.4 phosphate buffer

1 vs.				6 vs.			
2	*p*<0.05	*p*<0.01	*p*<0.001	7	*p*<0.01	*p*<0.001	*p*<0.01
3	*p*<0.01	*p*<0.01	*p*<0.01	8	*p*<0.001	*p*<0.001	*p*<0.001
4	*p*<0.01	*p*<0.01	*p*<0.01	9	*p*<0.001	*p*<0.001	*p*<0.001
5	*p*<0.001	*p*<0.001	*p*<0.001	10	*p*<0.001	*p*<0.001	*p*<0.001
A	*p*<0.001	*p*<0.001	*p*<0.001	A	*p*<0.001	*p*<0.001	*p*<0.001
B	*p*<0.001		*p*<0.001	B	*p*<0.001	*p*<0.001	*p*<0.001
2 vs.				7 vs.			
3	*p*<0.01	*p*<0.01	*p*<0.01	8	*p*<0.001	*p*<0.001	*p*<0.001
4	*p*<0.01	*p*<0.01	*p*<0.01	9	*p*<0.001	*p*<0.001	*p*<0.001
5	*p*<0.001	*p*<0.001	*p*<0.001	10	*p*<0.001	*p*<0.001	*p*<0.001
A	*p*<0.001	*p*<0.001	*p*<0.001	A	*p*<0.001	*p*<0.001	*p*<0.001
B	*p*<0.001	*p*<0.001	*p*<0.001	B	*p*<0.001	*p*<0.001	*p*<0.001
3 vs.				8 vs.			
4	ns	ns	ns	9	ns	ns	ns
5	*p*<0.001	*p*<0.001	*p*<0.001	10	*p*<0.001	*p*<0.001	*p*<0.001
A	ns	ns	ns	A	*p*<0.001	*p*<0.001	*p*<0.001
B	*p*<0.05	*p*<0.05	*p*<0.05	B	*p*<0.001	*p*<0.001	*p*<0.001
4 vs.				9 vs.			
5	*p*<0.001	*p*<0.001	*p*<0.001	10	*p*<0.001	*p*<0.001	*p*<0.001
A	ns	ns	ns	A	*p*<0.001	*p*<0.001	*p*<0.001
B	*p*<0.05	*p*<0.05	*p*<0.05	?B	*p*<0.001	*p*<0.001	*p*<0.001
5 vs.				10 vs.			
A	*p*<0.001	*p*<0.001	*p*<0.001	A	*p*<0.001	*p*<0.001	*p*<0.001
B	*p*<0.001	*p*<0.001	*p*<0.001	B	*p*<0.001	*p*<0.001	*p*<0.001
A vs B	ns	ns	ns				

ns, non–significant.

Kinetics of drug release from the tablets were calculated and evaluated according to zero-order, first-order and Higuchi kinetic models (24, 25). Drug release according to the first-order kinetic model is explained by the following relationship:

(b) $$\mathrm{logA}=\mathrm{kt}/2.303+{\mathrm{logA}}_{0}$$

where A is the amount of drug left in the matrix tablet; A_0_ is the initial amount of drug in the matrix tablet; k is the first-order release constant and t is the time.

Higuchi kinetic model (square root of time relationship) is explained with two relationships for homogenous and heterogenous matrix systems in Equations c and d, respectively:

(c) $$Q={\left[\mathrm{Dt}\left(2A-C_{S}\right)C_{S}\right]}^{1/2}$$

(d) Q=D ε/τ t2A−CSCS1/2

where Q is the cumulative amount of drug released in time t per unit surface area; D denotes the drug diffusion coefficient in the matrix phase; C_S_ is the drug solubility in the dissolution medium; ɛ and τ are the porosity and tortuosity factors in the matrix tablet, respectively; and A is the total amount of drug present in the matrix tablet.

**Figure 7:**
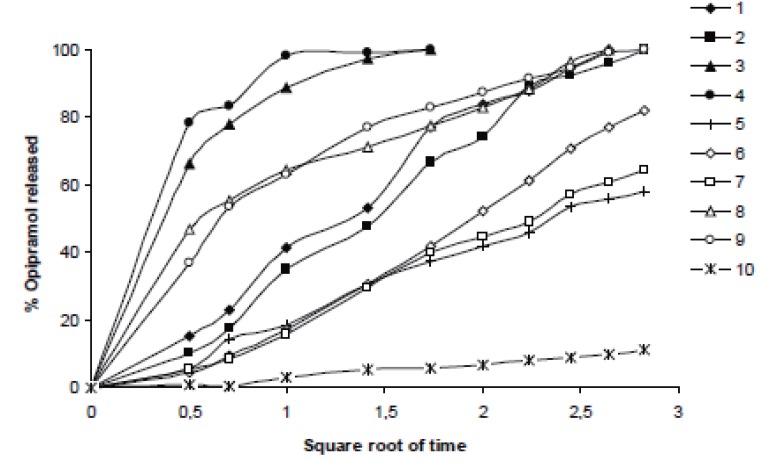
Higuchi release profiles of the related formulations in distilled water according to the Table 3.

**Figure 8:**
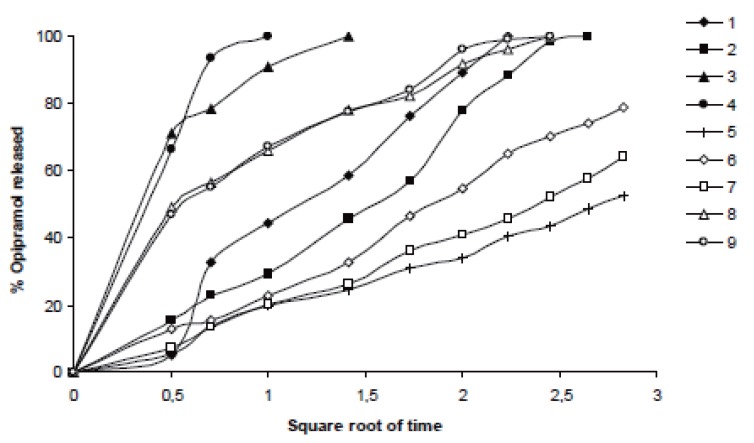
Higuchi release profiles of the related formulations in pH1.2 HCl buffer solution according to the Table 3.

**Figure 9:**
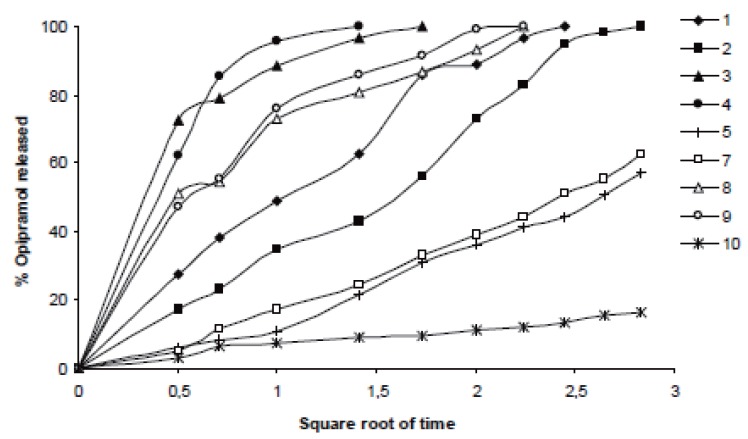
Higuchi release profiles of the related formulations in pH7.4 phosphate buffer solution according to the Table 3.

It was determined that matrix tablets generally released the drug according to the Higuchi kinetic model in water, pH1.2 HCl buffer and pH7.4 phosphate buffer solutions, but just in two cases where Formulations 6 and 10 (containing 20% HPC and C941, respectively) studied in phosphate buffer and HCl buffer solutions, drug release showed the zero-order model. None of them met the first-order model (Table 3 and Figures 7-9).

**Table 3:** Kinetic evaluation of drug release from the matrix tablets in various dissolution mediums according to zero-order, first-order and Higuchi kinetic models

Formulations	Distilled water			pH1.2 HCl buffer			pH7.4 phosphate buffer		
	Zero	First	Higuchi	Zero	First	Higuchi	Zero	First	Higuchi

1	0.8769	0.5166	0.9790	0.9234	0.5667	0.9740	0.8665	0.4231	0.9803
2	0.9178	0.5686	0.9854	0.9631	0.5704	0.9827	0.9484	0.5371	0.9851
3	0.5123	0.2903	0.9199	0.5433	0.0354	0.9778	0.4729	0.2791	0.9678
4	0.4000	0.2645	0.7938	0.7206	0.5341	0.8227	0.5515	0.3483	0.7881
5	0.9270	0.5941	0.9917	0.9371	0.5833	0.9894	0.9748	0.7009	0.9882
6	0.9794	0.7035	0.9957	0.9547	0.5850	0.9913	0.9922	0.6686	0.9865
7	0.9493	0.6684	0.9944	0.9641	0.6100	0.9936	0.9736	0.6562	0.9931
8	0.7131	0.2964	0.9895	0.6974	0.3005	0.9901	0.6760	0.3048	0.9669
9	0.7065	0.3109	0.9427	0.7151	0.3106	0.9762	0.6765	0.1215	0.9358
10	0.9550	0.7571	0.9845	0.9901	0.8152	0.9888	0.8918	0.6188	0.9629

Values are determination coefficients (r^2^) and best fits are bold.

As a result, Formulations 5 and 10 (containing C941 at 10% and 20% concentrations, respectively) were observed to release drug significantly slower compared to the other formulations as can be seen in Table 2. In case of cellulose derivatives, HPMC led slower drug release from the tablets compared with HPC in all the dissolution mediums. The tablets containing NaAlg and XG released drug similarly to the commercial tablets (especially in case of their 10% use) and it was concluded that they were not suitable for sustaining drug release in our study. Matrix tablets released OP according to the Higuchi kinetic model in all the mediums except Formulations 6 and 10, respectively. Their drug release showed the zero-order model in phosphate buffer and HCL buffer solutions.

In conclusion, Carbopol=941, HPMC and HPC can be suggested to prepare matrix tablets of OP which can be suitable for patient compliance and for reducing intoxication risk in long-term therapy. In addition, there are no risks in usage of the polymers employed in this study. HPMC and HPC have been reported to be safe according to human and animal feeding studies. They are widely used as excipients in oral pharmaceutical formulations and food products. They are generally regarded as nontoxic and nonirritant materials although excessive oral consumption may have laxative effect. In case of Carbopol=941, acute oral toxicity studies in animals demonstrated that it had low toxicities when ingested. Its LD_50_ (rat, oral) was reported as >1 g/kg (26).
